# Pathogenic Hantaviruses, Northeastern Argentina and Eastern Paraguay

**DOI:** 10.3201/eid1308.061090

**Published:** 2007-08

**Authors:** Paula Padula, Valeria P. Martinez, Carla Bellomo, Silvina Maidana, Jorge San Juan, Paulina Tagliaferri, Severino Bargardi, Cynthia Vazquez, Norma Colucci, Julio Estévez, María Almiron

**Affiliations:** * Instituto Nacional de Enfermedades Infecciosas ANLIS “Dr. Carlos. G. Malbrán,” Buenos Aires, Argentina; † Hospital de Enfermedades Infecciosas “Francisco J. Muñiz,” Buenos Aires, Argentina; ‡ Hospital de Pediatría de Posadas, Misiones, Argentina; § Universidad Nacional de Misiones, Posadas, Argentina ¶Laboratorio Central de Salud Pública, Asunción, Paraguay; # Ministerio de Salud Pública Provincial, Misiones, Argentina; ** Instituto de Investigaciones en Ciencias de la Salud, Asunción, Paraguay

**Keywords:** Hantavirus, Paraguay, Argentina, rodents, sigmodontine, Andes virus, Juquitiba virus, *Akodon*, Limoy Reserve, Misiones, dispatch

## Abstract

We describe the first, to our knowledge, cases of hantavirus pulmonary syndrome in northeastern Argentina and eastern Paraguay. Andes and Juquitiba (JUQ) viruses were characterized. JUQV was also confirmed in 5 *Oligoryzomys nigripes* reservoir species from Misiones. A novel *Akodon*-borne genetic hantavirus lineage was detected in 1 rodent from the Biologic Reserve of Limoy.

Members of the genus *Hantavirus* (family *Bunyaviridae*) are commonly transmitted to humans through rodents and may cause 2 severe human diseases: hemorrhagic fever with renal syndrome and hantavirus pulmonary syndrome (HPS) (*1*). The number of recognized human cases and the number of distinct hantavirus genotypes identified have increased during recent years in Argentina and in the 3 southern HPS-endemic states of Brazil (*2*,*3*). Six pathogenic Andes virus (ANDV) lineages that cause HPS have so far been found to circulate in Argentina in 3 HPS-endemic areas: Oran and Bermejo (BMJ) in the north; Lechiguanas (LEC), Hu39694, and AND Cent (Central) Plata in the central provinces; and ANDV in the South (*4*–*7*).

Different hantavirus genetic lineages associated with HPS cases were reported in Brazil, such as Juquitiba virus (JUQV), Castelo dos Sonhos virus (CASV), and Araraquara virus (ARAV) (*8*). Recently, pathogenic hantaviruses from Parana, southern Brazil, have been reported to belong to the same clade as the *Oligoryzomys nigripes*–associated strains (*9*). In western Paraguay, cases were associated with Laguna Negra virus (LNV) from *Calomys laucha* (*10*). In northern Argentina and Bolivia, LNV was obtained from *C. callosus* (*11*,*12*). No cases have been reported in other areas of Paraguay, to our knowledge, until this study.

We describe 3 HPS cases that occurred in northeastern Argentina, Misiones Province, which borders 2 other hantavirus-endemic countries, Brazil and Paraguay. We also describe what we believe to be the first case that occurred in eastern Paraguay and analyzed rodents captured in Misiones and characterized a novel genetic hantavirus lineage from the Biologic Reserve of Limoy in eastern Paraguay.

## The Study

**Figure 1:**
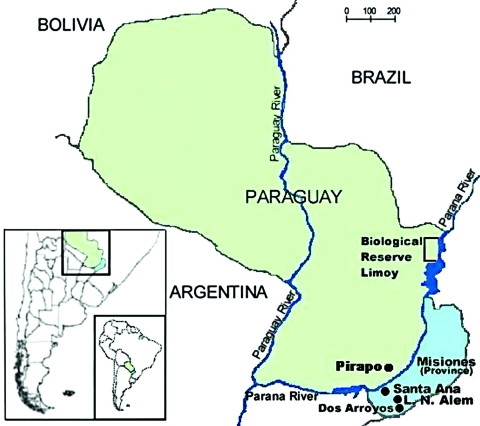
Misiones Province, Argentina, and eastern Paraguay, where cases of hantavirus pulmonary syndrome have occurred and rodents were trapped for testing.

Three HPS cases were confirmed in Misiones Province, Argentina, in the following patients: a 14-year-old boy from Santa Ana in November 2003, a 28-year-old man from Leandro N. Alem in December 2003, and a 12-year-old boy living in the Dos Arroyos locality of Alem city. In January 2005, HPS was confirmed in a 19-year-old girl living in Pirapo, a rural area of Itapua Department, Paraguay. Figure 1 shows the approximate geographic location of exposure sites for patients included in this study. Clinical manifestations in the 4 patients studied were similar to those reported for ANDV infections: fever, myalgia, headache, and vomiting, soon followed by pulmonary edema. Thrombocytopenia and hemoconcentration were reported, renal involvement was minimal, neither oliguria nor renal failure was observed in any case-patient, but all case-patients showed petechiae. The 4 HPS case-patients had immunoglobulin M (IgM) and IgG antibodies to ANDV N recombinant protein by ELISA (*13*) and all survived. These cases led us to investigate reservoirs for hantaviruses in Misiones by using Sherman live-capture traps (H.B. Sherman Traps, Tallahassee, FL, USA). A total of 59 rodents were trapped at 2 study sites in Misiones, where *O. nigripes* was the most frequently captured rodent (42 specimens), followed by *Akodon montensis* (11 specimens). The rodent species was identified by morphologic features, in particular, qualitative external and cranial characteristics. Animals were tested for ANDV IgG antibodies (Table 1). The capture done in August 2005 in Santa Ana and Leandro N. Alem cities found 5 (11.9%) ANDV-positive *O. nigripes* rodents of 42 tested from this species.

**Table 1:** Sigmodontine rodents captured and surveyed for antibodies to Andes hantavirus and viral RNA, northeast Argentina and eastern Paraguay*

Location	Period	Species	No.	Seropositive rodents, no (%)	RT-PCR– positive rodents
Caaguazu, Paraguay	Spring 2000	*Akodon* sp.	1	0	
		*Calomys callosus*	1	0	
Caaguazu, Paraguay	Summer 2001	*Holochilus brasiliensis*	4	0	
		*C. callosus*	1	0	
Central Paraguay	Summer 2001	*Oryzomys buccinatus*	2	0	
Reserve Limoy, Paraguay	Winter 2001	*A. cursor*	7	1 (14.3)	1
		*C. callosus*	2	0	0
Caaguazu, Paraguay	Spring 2001	*A. cursor*	8	0	
		*A. nigrita*	1	0	
Reserve Limoy, Paraguay	Summer 2002	*A. cursor*	5	0	
Leandro N. Alem, Misiones, Argentina	Autumn 2004	*Oligoryzomys* sp.	9	0	
		*A. cursor*	3	0	
		*O. nigripes*	1	0	
		*O. flavescens*	1	0	
		*H. brasiliensis*	1	0	
Santa Ana Misiones, Argentina	Winter 2005	*O. nigripes*	28	3 (10.7)	3
		*A. montensis*	8		
		*O. flavescens*	1		
Leandro N. Alem, Misiones, Argentina	Winter 2005	*O. nigripes*	14	2 (14.3)	2
		*A. montensis*	3	0	
		*O. flavescens*	3	0	
		*C. laucha*	2	0	

*RT, reverse transcription.

Before we discovered the case in Itapua, we had conducted a serosurvey of rodents in 3 departments in eastern Paraguay (Table 1). Fifty-one rodent specimens were collected, of which 32 were sigmodontine rodents and 20 were *Akodon cursor*. Only 1 *A. cursor* rodent obtained from the Limoy Biologic Reserve, Paraguay, was seropositive. Because of the diversity of akodontine rodents, precise diagnosis, based solely on morphometric characteristics, is not always possible. To confirm the morphologic identification of the *A. cursor* rodent, we compared the mitochondrial control region (fragment of 245 nt) with that of an *A. montensis* rodent used as a control. Mitochondrial DNA sequencing of *A. cursor* from Limoy found higher identity (94%) with an *A. cursor* from Paraguay (AF296264) than with *A. montensis* (90%). Positive voucher specimens were archived at the Museo Argentino de Ciencias Naturales and at the University of Buenos Aires.

Amplification by reverse transcription–PCR was performed on the 4 human blood samples and on the lung tissues of the 6 seropositive rodents. Initially, a substantial portion of the nucleoprotein N coding region of the S segment (nt 50–954) and different fragments of the encoding region of the M segment: G1 glycoprotein (nt 41–443), G1–G2 glycoprotein (nt 1,728–1,976), and G2 glycoprotein (nt 2,715–2,941) were amplified and subsequently sequenced.

Comparison of the viral 905-nt N fragment sequence from case-patient 1 showed the highest degree of identity, ≈90%, with LEC (Table 2). The strains from case-patients 2 and 3 showed little genetic variation between them and were ≈95% identical to ARAV from Parana city, Brazil (*12*), in the same fragment. Comparison of a G1–G2 fragment available for JUQV strain (nt 1,867–1,976) with that from case-patient 2 showed a 93.6% identity. The G2 fragment from case-patient 3 was 95.6% identical to that of JUQV. Thus, these results suggest that the strains from case-patients 2 and 3 are JUQV, although they demonstrate that the strain called Araucaria would also be JUQV.

**Table 2:** Comparison of the nucleotide (first) and amino acid (second) sequences of the 904-nucleotide region of the N gene ORF among human and rodent hantavirus strains from South America with viruses from Misiones, Argentina, and eastern Paraguay*

	Case 1	Cases 2 ,3	Case Itapua	*Akodon cursor* Limoy	*O. nigripes*	LEC	Hu 39694	AND Central Plata	ORN	BMJ	AND	ARA†	CAS†	JUQ	LN	HTN 007	RIME	ANAJ	RIOM	MAP	Choclo	SN
Case 1		82.2	91.2	74.1	82.1	96.5	87.8	93.2	87.5	92.3	83.8	82.3	86.2	82.5	77.6	80.6	76.2	80.1	80.1	77.0	77.7	76.4
Cases 2, 3	94.7		84.2	75.8	98.5	81.9	82.4	82.1	83.2	83.1	82.3	84.0	84.0	95.1	80.1	78.9	76.8	80.0	79.2	80.4	78.3	76.7
Case Itapua	99.3	94.0		74.0	84.0	90.8	87.7	91.4	88.2	93.5	83.5	83.5	86.3	84.9	78.7	80.1	77.0	81.0	79.5	79.2	77.7	75.9
*Akodon cursor* Limoy	86.1	85.4	85.7		75.7	74.8	75.5	74.5	75.4	74.7	75.7	77.2	77.8	75.9	74.7	77.4	76.8	76.9	76.9	76.4	75.4	73.9
*Oligoryzomys nigripes*	94.3	99.7	93.7	85.0		82.0	81.6	81.9	82.9	82.4	82.1	83.8	84.0	95.1	79.8	79.3	76.7	80.0	79.3	80.1	78.0	76.2
LEC	99.7	94.3	99.0	85.7	94.0		87.2	92.6	87.2	91.7	84.8	82.6	87.1	83.0	78.8	80.8	76.9	80.2	80.6	78.7	77.5	76.1
Hu39694	99.7	95.0	99.0	86.0	94.7	99.3		88.5	87.9	88.3	83.9	82.9	82.6	82.0	79.2	78.8	78.0	79.5	78.8	78.3	78.9	75.0
AND Central Plata	100.0	94.7	99.3	86.0	94.3	99.3	99.3		87.2	92.0	82.9	83.4	85.4	82.8	78.9	80.0	76.5	80.0	79.9	78.9	77.8	76.5
ORN	98.3	96.0	97.7	85.4	95.7	98.0	98.7	98.0		87.3	83.8	84.3	84.9	83.4	78.9	80.8	77.4	80.0	80.7	79.1	78.5	76.5
BMJ	100.0	94.7	99.3	86.0	94.3	99.7	99.7	99.7	98.3		84.3	83.2	86.5	83.5	79.0	79.9	77.7	79.9	79.8	77.7	77.7	76.8
AND	95.7	94.7	95.0	85.7	94.3	95.4	96.0	95.4	95.7	95.7		85.1	85.5	83.4	78.8	79.0	79.5	79.2	78.9	79.3	78.3	75.7
ARA†	95.3	93.9	94.9	89.2	93.5	95.3	95.8	94.9	96.3	95.3	96.3		81.8	83.7	81.7	80.7	78.9	80.6	82.3	81.7	81.0	79.2
CAS†	98.1	94.9	97.7	91.6	94.4	98.1	97.7	97.6	97.2	98.1	98.1	94.4		84.8	80.4	81.2	79.9	81.8	81.2	81.2	79.8	79.8
JUQ	94.7	100.0	94.0	85.4	99.7	94.4	95.0	94.4	96.0	94.7	94.7	93.9	94.9		80.6	79.1	77.2	80.7	79.0	80.7	77.9	76.4
LN	86.7	87.4	86.0	85.0	87.0	86.4	87.0	86.4	87.0	86.7	87.7	90.7	91.6	87.4		82.0	79.2	80.2	83.1	77.7	78.5	75.4
HTN 007	88.0	88.4	87.7	88.4	88.0	87.7	88.4	87.7	88.4	88.0	89.4	90.7	91.6	88.4	91.7		83.1	85.8	87.1	81.0	78.5	74.6
RIME	88.4	87.7	88.0	88.7	87.4	88.0	88.4	88.4	88.4	88.4	88.4	90.6	92.1	87.7	91.0	96.3		83.4	82.2	79.0	76.6	74.8
ANAJ	87.7	89.0	87.4	88.0	88.7	87.4	88.0	87.7	88.0	87.7	89.0	90.2	91.1	89.0	91.4	97.3	95.7		85.6	79.7	76.8	75.5
RIOM	88.0	87.4	87.4	87.7	87.0	88.4	88.4	87.7	88.4	88.0	88.7	90.7	91.6	87.4	91.7	97.7	94.3	95.7		81.2	77.6	75.3
MAP	89.4	88.4	89.4	85.7	88.0	89.7	89.0	89.0	88.7	89.4	89.4	92.5	94.4	88.4	87.0	89.7	88.7	89.4	89.7		77.6	75.3
Choclo	88.7	86.4	88.4	86.0	86.0	88.4	88.4	88.4	87.4	88.7	88.0	93.9	93.9	86.4	85.4	88.4	86.3	87.4	87.0	87.7		74.2
SN	85.4	84.1	85.0	84.0	84.0	85.1	85.1	85.1	84.7	85.4	83.7	89.3	91.1	84.1	82.4	82.7	83.0	82.4	82.4	83.7	85.7	

*ORF, open reading frame; LEC, Lechiguanas virus; ORN, Oran VIRUS; BMJ, Bermejo virus; AND, Andes virus; ARA, Araraquara virus; CAS, Castelo dos Sonhos virus; JUQ, Juquitiba virus; LN, Laguna Negra virus; RIME, Rio Mearim virus; ANAJ, Anajatuba virus; RIOM, Rio Mamoré virus; MAP, Maporal virus; SN, Sin Nombre virus. †Comparison done on 636 nt.

Sequences from the 5 positive *O. nigripes* from Misiones showed little variability between them and the N fragments were 98.3% identical to those in strains from case-patients 2 and 3. The case-patient from Itapua, Paraguay, showed the greatest nucleotide identity (93.5%) with BMJ lineage, in the N fragment; identity at the amino acid level was 99.3%.

Another different strain was obtained from the *A. cursor* rodent captured in Limoy Reserve; this was the most distinct strain. The highest degree of identity exhibited was ≈77% (Table 2). The G1 fragment from this strain was compared with the closest related hantavirus and showed 67% identity with strains isolated from *O. nigripes*, case-patients 2 and 3, and LNV.

**Figure 2:**
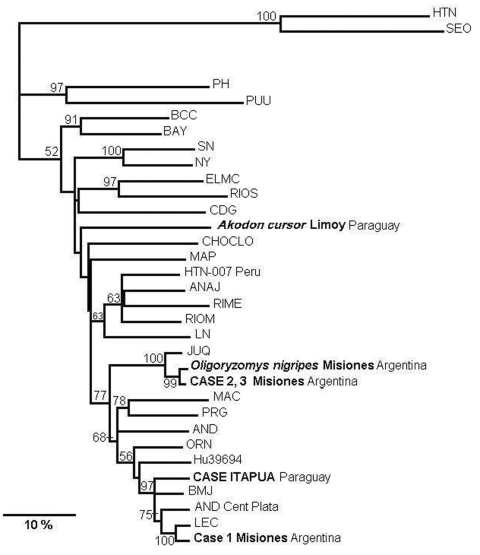
Phylogenetic relationships among the nucleotide sequences of the N protein of different hantaviruses from North America. A maximum parsimonious phylogenetic tree was generated on the basis of nucleotide sequence differences in the 904-nt region of the N gene open reading frame, which is available for South American strains by using PHYLIP version 3.57c. Bootstrap values >50%, obtained from 1,000 replicates of the analysis, are shown for the branch points. Lengths of the horizontal branches are proportional to the nucleotide step differences. The strain sequences under study in this paper are in italics. The following published S-segment sequences were included in the analysis (GenBank accession no.): Hantaan (HTN; U37768), Seoul (SEO; AB027522), Prospect Hill (PH; Z49098), Puumala (PUU; X61035), Black Creek Canal (BCC; L39949), Bayou (BAY; L36929), Sin Nombre (SN; L25784), New York (NY; U36801), El Moro Canyon (ELMC; U11427), Río Segundo (RIOS; U18100), Caño Delgadito (CDG; AF000140), Choclo (CHOCLO; DO285046), Maporal (MAP; AY267347), HTN-007 Perú (HTN-007 Perú. AF133254), Anajatuba (ANAJ; DQ451829), Rio Mearim (RIME; DQ451828), Río Mamoré Bolivia (RIOM; U52136), Laguna Negra (LN; AF005727), Araucaria (JUQ; AY740633), Maciel (MAC; AF0482716), Pergamino (PRN; 482717), Andes (AND; AF324902), Oran (ORN; AF028024), Hu39694 (Hu39694; AF482711), Bermejo (BMJ; AF482713), Lechiguanas (LEC; AF482714).

The N-encoding sequences were further subjected to phylogenetic analysis. All virus sequences from Misiones and the Itapua case form a monophyletic group together with ANDV lineages, nonpathogenic Pergamino (PRG) and Maciel (Figure 2), and CASV and ARAV (done in a 643-nt parsimonious tree, data not shown). The other South American clade was formed with Rio Mearim virus and Anajatuba virus from Brazil, together with LNV, HTN virus 007, and Rio Mamoré virus from Paraguay, Peru, and Bolivia, respectively, with a moderate support of 63%. Sequences from case-patients 2 and 3 from Misiones grouped together with JUQV (Araucaria) to form a separate clade, since they were from the most divergent strains. The sequence from case-patient 1 grouped with LEC lineage from Central Argentina. Different alignment parameters and phylogenetic methods produced the same results in trees with similar topology; however, bootstrap supports were moderate or low level for some lineages. The sequence from the *A. cursor* rodent was a quite genetically distinguishable virus lineage, separated and apart from Choclo and Maporal viruses.

## Conclusions

HPS is an emerging disease in South America, and investigations strengthen the belief that the disease is underestimated. Despite being surrounded by HPS-endemic countries, Misiones Province had no reported HPS cases until 2003. Furthermore, HPS cases have not been documented in eastern Paraguay. Two pathogenic hantaviruses that cause HPS have so far been proven to circulate in Misiones: LEC and JUQV. We confirmed *O. nigripes* as the reservoir species associated with 2 JUQV cases in Misiones. In eastern Paraguay, Itapua Department, BMJ lineage produced HPS. We have already reported a BMJ case in Bolivia (*7*). LEC was characterized originally from an *O. flavescens* mouse trapped in the Rio de la Plata River, an area where several HPS cases had occurred (*6*).

Species of *Akodon* are found throughout South America. To our knowledge, no *Akodon*-borne hantavirus has been reported to be associated with cases in South America. *A. azarae* is the most abundant sigmodontine species widely distributed in rural and peridomestic habits of central Argentina. In Buenos Aires Province, PRG was characterized in *A. azarae* populations. Despite the absence of reported HPS cases associated with this species in the studied area, a high seroprevalence, ≈10%, has been detected (*14*). We characterized a distinct *Akodon*-borne hantavirus at the Biologic Reserve of Paraguay, although we did not investigate whether this virus can produce illness. A prior study analyzed a collection of sigmodontine rodents from the major biomes of Paraguay where 1 *A. montensis* and 2 *O. nigripes* were positive for viral RNA (*15*). Precise identification of source populations in the reservoir and collection of quantitative data on their relative contribution to hantavirus transmission will be essential for disease control in the 3-country frontier.
